# Review of the Latest Percutaneous Devices in Critical Limb Ischemia

**DOI:** 10.3390/jcm7040082

**Published:** 2018-04-14

**Authors:** Leila Haghighat, Sophia Elissa Altin, Robert R. Attaran, Carlos Mena-Hurtado, Christopher J. Regan

**Affiliations:** 1 Department of Internal Medicine, Yale-New Haven Hospital, New Haven, CT 06520 USA; leila.haghighat@yale.edu; 2 Section of Cardiovascular Medicine, Department of Internal Medicine, Yale University School of Medicine, New Haven, CT 06520, USA; elissa.altin@yale.edu (S.E.A.); robert.attaran@yale.edu (R.R.A.); carlos.mena-hurtado@yale.edu (C.M.-H.)

**Keywords:** critical limb ischemia, peripheral artery disease, percutaneous transluminal angioplasty, drug-coated balloons, bare metal stents, drug-eluting stents, atherectomy, cryoplasty, embolic protection devices

## Abstract

Critical limb ischemia (CLI) is a terminal stage of peripheral arterial disease that, in the absence of intervention, may lead to lower extremity amputation or death. Endovascular interventions have become a first-line approach to the management of CLI and have advanced considerably within the past decade. This review summarizes the types of percutaneous devices and the techniques that are available for the management of CLI and the data supporting their use. These include devices that establish and maintain vessel patency, including percutaneous transluminal angioplasty, drug-coated balloons, bare metal stents, drug-eluting stents, bioresorbable vascular scaffolds, and atherectomy; devices that provide protection from embolization; and, cell-based therapies. Additionally, ongoing trials with important implications for the field are discussed.

## 1. Introduction

Critical limb ischemia (CLI) is severe arterial insufficiency in the lower extremities, resulting in pain with or without tissue loss for more than two weeks. These symptoms are confirmed with objective hemodynamic markers of poor perfusion, including the ankle/brachial index (ABI), toe pressures, transcutaneous oxygen pressures, and skin perfusion pressures. Guidelines vary on the cutoffs of these indices that define CLI, although there are interdisciplinary consensus definitions [1]. CLI affects approximately 1% of the adult population. Up to 10% of patients with symptomatic peripheral arterial disease (PAD) and an additional 5–10% of patients with asymptomatic PAD progress to CLI over a five-year period [2]. CLI represents a terminal stage of PAD, corresponding to Rutherford Class 4–6 and Fontaine Class 3–4 [3]. These two classification schemes stratify patients by clinical symptomology and can be used to determine patient’s risk of amputation and likelihood of benefit from revascularization. These schemes address arterial perfusion alone, however prompting the development of the Threatened Limb Classification system [WIfI] that is proposed by the Society for Vascular Surgery incorporating wound extent and concomitant foot infection [4].

There is significant morbidity and mortality associated with CLI, which underscores its clinical importance. Projected amputation risks four years after index hospitalization for CLI range between 12.1–67%, depending on Rutherford classification [5]. A two-year mortality rate of 41% has been reported, which is accounted for mostly by cardiovascular disease [6]. In the PREVENT III trial more than 40% of patients with CLI had advanced coronary artery disease [7].

Open surgical techniques previously dominated the treatment of CLI. However, several limitations to surgery are recognized: high peri-operative risk due to concomitant medical illness, anatomic challenges of obtaining adequate native grafts, inadequate targets for distal anastomosis, and graft closure. In light of these limitations, endovascular approaches have been developed that are now considered to be a first-line approach for CLI treatment in properly selected patients. Rates of surgical revascularization have significantly declined, from 13.9% in 2003 to 8.8 % in 2011, while endovascular revascularization has increased, from 5.1% to 11% during the same period in the United States [8]. Given the increased role of endovascular treatment of CLI, this review describes the current percutaneous devices that are available for the management of CLI with a particular focus on infrapopliteal revascularization, which is often required in the management of CLI and highlights the ongoing clinical trials in the field that are anticipated to provide data-driven changes in management for this morbid disease.

## 2. Percutaneous Devices for Vessel Patency

### 2.1. Percutaneous Transluminal Angioplasty (PTA)

PTA was compared directly with surgical revascularization in the 2005 BASIL study [9]. A total of 452 patients from several sites across the UK were recruited into the study and were randomized into either a surgery-first or angioplasty-first approach. At the conclusion of the study, there was no difference in the primary outcome of amputation-free survival (AFS) at 12 months and three years following intervention. In the short-term, a surgery-first approach was associated with earlier morbidity, longer hospital stay, higher costs, and greater use of ICU care. However, after 12 months the angioplasty-first approach had higher rates of re-intervention and after two years higher rates of amputation and death. PTA has largely been replaced by use of drug coated balloons or stents in the femoral and popliteal arteries given the higher rates of restenosis, but is still frequently employed in the infrapopliteal vessels.

### 2.2. Drug-Coated Balloons (DCBs)

Above-the-knee DCBs were studied in the 2015 IN.PACT SFA trial [10]. This multi-center international trial randomized 331 patients to either DCB placement or PTA. At 12 months, patients who underwent DCB placement had higher rates of patency (8.2 vs. 52.4, *p* < 0.001). Below-the-knee DCBs were studied in the 2013 DEBATE-BTK trial [11]. A single-center study of 132 patients that were randomized in a 2:1 fashion to receive either DCB or PTA, the trial’s primary outcome favored the DCB group. In particular, the DCB group had lower rates of restenosis at 12 months (27% vs. 75%, *p* < 0.001), along with lower rates of revascularization and target vessel occlusion, with no difference in AFS. A larger study of below-the-knee DCBs was performed in the 2014 IN.PACT DEEP trial [12]. The trial was a multi-center study of 358 patients who were randomized 2:1 to receive either DCB or PTA. There was no difference in the co-primary endpoints of target lesion revascularization (TLR) (9.2% vs. 13.1%, *p* = 0.291) and late lumen loss (0.61 mm vs. 0.62 mm, *p* = 0.95). A higher rate of amputation was noted in the DCB group, though not being statistically significant, and notably, at baseline, the DCB group had higher rates of impaired inflow and prior target limb revascularization. Drug coated balloons have been heavily adopted in the femoral and popliteal arteries given superior results against balloon angioplasty. Drug coated balloons are also favored over stenting in situations in which significant dissection or vessel recoil are absent. Drug coated balloons are not regularly utilized in the tibial vessels given the failure to demonstrated superiority over PTA and the safety signal of more frequent amputations. 

### 2.3. Bare Metal Stents (BMS)

BMS attempt to overcome the shortcomings of PTA, especially vessel recoil, dissection, and restenosis. The 2012 XCELL trial demonstrated the utility of BMS [13]. All 120 patients in this multi-center study underwent the placement of a self-expanding nitinol stent. The primary endpoint looked at 12-month AFS, which was 78.3%. 12-month TLR was 29.9% with a wound-healing rate of 54.4%. Bare metal stents play an important role in the revascularization of the femoral and popliteal arteries, especially in the setting of significant dissection following angioplasty and the opening of chronic total occlusions utilizing dissection and reentry. Their use is limited by stent fracture, particularly at areas of flexion within the leg and concerns regarding the difficulty of treating subsequent in stent restenosis. In the tibial vessels, stents are less often employed given the typically long length of lesions and the small vessel size that make them prone to restenosis.

### 2.4. Drug-Eluting Stents (DES)

The 2010 PaRADISE trial was a non-randomized trial of 106 patients who received either sirolimus- or paclitaxel-containing DES in below the knee vessels with the aim of demonstrating the efficacy and safety of DES [14]. There were no procedural deaths, and after three years, the amputation rate was 6%, survival rate was 17%, and AFS rate was 68%. The 2012 ACHILLES trial demonstrated that restenosis was lower in patients that were randomized to a sirolimus DES when compared to PTA in below the knee vessels (22.4% vs. 41.9%, *p* = 0.019) [15]. Additionally, there was greater vessel patency in the DES group with similar rates of death, revascularization, and death between the two groups. The 2012 DESTINY trial showed the benefit of an everolimus DES when compared to BMS in below the knee vessels, with higher patency rates at 12 months, as defined by stenosis that was less than 50% on angiogram (85% vs. 54%, *p* = 0.0001). Among secondary endpoints, though, the TLR rate was notably higher in the everolimus group (91% vs. 66%, *p* = 0.001) [16]. The 2012 YUKON-BTK study was a multi-center randomized trial of 161 patients that compared a sirolimus DES to BMS in below the knee vessels and found a higher event-free survival rate among patients with the DES (65.8% vs. 44.6%, *p* = 0.02), in addition to lower amputation rates and TLR rates [17]. Drug eluting stents, notably the paclitaxel eluting Zilver PTX, have demonstrated superiority over angioplasty, but their use is largely confined to the femoral and popliteal arteries. Below the knee the use of drug eluting stents is largely confined to bailout following angioplasty.

### 2.5. Bioresorbable Vascular Scaffold (BVS)

Stent placement may affect the arterial vascular wall by preventing auto-regulation and adaptive remodeling. Additional risks of stent placement include stent fracture and malapposition. Bioresorbable scaffolds have been created as a means to overcome these limitations. A study of 33 patients in Australia that were treated with bioresorbable vascular scaffolds showed restenosis rates of 6% after a mean follow-up of 12 months [18]. TLR rates at 12 and 24 months were 96% and 84.6%, respectively, with wound healing in 64% of patients, and no amputations. The 2016 ESPRIT I trial studied the safety of a drug-eluting BVS. An everolimus-eluting scaffold was placed in 35 patients, with no procedure or device-related death or amputation within two years [19]. AFS rate was 100% with a restenosis rate of 16.1% and target lesion revascularization of 11.8%. Bioresorbable scaffolds remain investigational and are not routinely employed outside of clinical trials. 

### 2.6. Atherectomy

Atherectomy devices include laser, directional, rotational, and orbital. Laser atherectomy involves the delivery of a burst of ultraviolet energy in a short pulse that ablates tissue directly in contact with the catheter, with minimal surrounding thermal injury. In the international 2006 LACI trial, 145 patients with CLI, who were adjudicated as poor candidates for peripheral artery bypass surgery, underwent laser atherectomy followed by PTA with optional stenting, which occurred in 45% of cases [20]. Overall, 86% of procedures were successful, with less than 50% residual stenosis, and the patency rate at six months was 93%. Directional atherectomy makes use of a cutting blade to debulk plaque. The 2014 DEFINITIVE-LE trial was a multi-center study of 800 patients that was looking at the safety and efficacy of directional atherectomy [21]. At 12 months, the primary endpoint of patency was 78% with the additional endpoint of 95% AFS. Peri-procedural adverse events included abrupt closure, embolization, and most commonly, perforation, which occurred in 5.3% of cases. The 2017 DEFINITIVE-AR trial was a multi-center study of 102 patients that were randomly assigned to either directional atherectomy with DCB or DCB alone [22]. The angiographic diameter of stenosis was 33.6% in the former group as compared to 36.4% in the latter, although the study was not powered to establish a significant difference. Rotational atherectomy uses a concentrically rotating burr to debulk plaque. This modality was studied prospectively in a multi-center study of 72 patients in whom angiographic success of less than 25% stenosis was attained in 77% of procedures with a 31% patency at 12 months [23]. Orbital atherectomy is performed with the DiamondBack 360° Orbital Atherectomy System (Cardiovascular Systems, Inc., St. Paul, MN, USA). This device utilizes an orbiting diamond-coated crown. In the 2014 COMPLAINCE 360° study, 50 patients were randomized to receive either orbital atherectomy with PTA or PTA alone [24]. At 12 months, there was no difference in freedom from TLR between the two groups (77.1% vs. 11.5%, *p* < 0.001), although patients who received PTA alone had higher rates of adjunctive stenting required during the initial procedure. 

Certain atherectomy devices have the additional capacity for aspirating debulked plaque to reduce the risk of embolization. One such device is the Jetstream Catheter (Boston Scientific, Marlborough, MA, USA), which provides active aspiration along with rotational and optionally directional atherectomy. While aspiration techniques have not been well studied exclusively in the CLI population, a study of the Jetstream Catheter in 172 patients with Rutherford Class 1–5 lower limb ischemia demonstrated success in 99% of cases with a one-year restenosis rate of 38.2% that was based on Duplex ultrasound [25]. The Phoenix catheter (Philips, Amsterdam, The Netherlands) utilizes an Archimedes screw design to debulk plaque and draw it into a waste bag. The safety and efficacy of the Phoenix catheter is currently under investigation in the multi-center EASE trial (NCT01541774). Use of atherectomy is commonly employed in heavily calcified vessels above and below the knee in order to improve luminal gain in response to angioplasty. The choice of a particular device is often dependent upon operator preference and device availability at particular institutions.

### 2.7. Cryoplasty

Cryoplasty delivers pressurized liquid nitrous oxide at −10 °C for 20 seconds via a balloon catheter, followed by a passive warming cycle. In the 2009 BTK Chill Trial, 108 patients were treated with cryoplasty with a primary endpoint of success, which was defined as less than 50% residual stenosis, attained in 97.3% of procedures and 12 months AFS of 85.2% [26]. There is insufficient current data to support the routine use of cryoplasty in the treatment of peripheral arterial disease.

### 2.8. Percutaneous Embolic Protection Devices (EPD)

Given the potential for distal embolization with atherectomy, embolic protection devices are commonly employed to minimize this risk. Embolized particles that are greater than 1 mm and 3 mm in a single dimension were detected in 58% and 12% of cases, respectively, in a single-center prospective study of 48 patients undergoing the endoluminal therapy of infra-aortic lesions with standard endovascular procedures [27]. Among EPDs is the Emboshield NAV6 filter (Abbott Vascular Inc., Chicago, IL, USA), which is a protective shield that can be combined with atherectomy. A study of 141 patients in the Excellence in Peripheral Artery Disease (XL-PAD) registry (NCT01904851) who underwent intervention with the Jetstream catheter with and without the additional use of the NAV6 filter were compared [28]. Those who also had the NAV6 filter used experienced longer procedure duration and longer fluoroscopy times, but lower rates of distal embolization (1.8% vs. 8%, *p* = 0.1). Another EPD that functions as a filter basket is the SpiderFX (Medtronic, Minneapolis, MN, USA). The 2008 PROTECT study was a prospective study of 40 patients who underwent treatment with angioplasty/stenting or atherectomy, and an EPD, either the EmboShield or SpiderFX [29]. Macroembolization occurred in 55% of cases, and macrodebris at least 2 mm in diameter occurred in 45% of cases, mostly among patients undergoing atherectomy.

### 2.9. Percutaneous Devices to Pass Chronic Total Occlusions (CTOs)

CTOs are commonly encountered in CLI and occur in up to 40% of cases with symptomatic PAD [30]. The most common method for crossing CTOs is wire escalation, in which wires of increasing diameter, tip loads, and hydrophobicity are used to cross the lesion. This method frequently utilizes the subintimal space to cross the lesion, but ultimately requires the reentry into the true lumen for the completion of a successful intervention. Reentering the true lumen can be performed with the wire or with the assistance of the Outback (Cordis, Milpitas, CA), which utilizes intravascular ultrasound to guide reentry. Retrograde access through the tibiopedal vessels may also facilitate the crossing of difficult CTOs, which are challenging to cross in antegrade fashion. There are a variety of devices that can be utilized to assist in crossing via the true lumen, but none have been specifically tested in CLI. These include devices with rotating tips designed to bore through plaque, such as the Wildcat/Kittycat (Avinger Inc., Redwood City, CA, USA) and True Path (Boston Scientific, Marlborough, MA, USA) devices. Others have blunt tips for microdissection, such as the Frontrunner (Cordis Endovascular, Miami, FL, USA) and Viance (Medtronic, Minneapolis, MN, USA). Still, others use high frequency vibrations propagated by the tip to disrupt plaque, such as the Safe Cross (Intraluminal Therapeutics, Carlsbad, CA, USA) and Crosser (Bard, Tempe, AZ, USA). The 2017 SUPERSUB study was a prospective, single center study of 34 CLI patients with CTOs who underwent placement of a BMS after subintimal crossing [31]. At 12 months, patency was attained in 94.1% of cases with freedom from TLR of 97.1%. Use of particular techniques or devices for crossing of CTOs has not been studied in randomized comparison trials, and so the use of particular devices depends upon operator preference and device availability.

### 2.10. Cell-Based Therapies

The injection of various cell stimulating factors that promote angiogenesis and bone marrow-derived pluripotent cells has been studied in the management of PAD. These include fibroblast growth factor (FGF) 1, vascular endothelial growth factor (VEGF), and hepatocyte growth factor (HGF). In pilot studies, these therapies have demonstrated efficacy in improving subjective and surrogate outcomes, like ABI, transcutaneous oxygen measurement, walking distance, and pain scores [32]. However, in larger and placebo-controlled studies, these have not proven efficacious [33]. The 2015 JUVENTAS trial randomized 160 patients to receive intra-arterial infusion of bone marrow-derived mononuclear cells or placebo [34]. At six months, no significant difference was found in safety outcomes, all-cause mortality, or secondary outcomes, including ABI and transcutaneous oxygen pressures. The 2011 TAMARIS trial showed no difference in the primary endpoint of reduction in amputation or death between patients that were randomized to intramuscular injections of FGF-1 as compared to placebo [35].

## 3. Ongoing Trials and Future Directions

The LIBERTY trial (NCT01855412) is an ongoing prospective, multi-center, observational clinical trial studying the acute and long-term clinical and economic outcomes of various FDA-approved endovascular interventions in PAD, including the stages of disease that qualify as CLI. Enrollment of over 1200 patients was completed in 2016 with the plan of following these patients for five years. Preliminary data from the trial has shown that as late as 12 months after endovascular intervention patients with Rutherford Classification 4–5 and 6 have an AFS rate of 96% and 91.7%, respectively. Additionally, after intervention these CLI patients show an improvement in Rutherford classification, wound healing, and quality of life measurements [36]. Several ongoing trials are looking at the outcomes of combining atherectomy with DCB. LUTONIX-BTK (NCT01870401) is one such multi-center trial of 480 patients with the primary outcome of patency and limb salvage at six months. BEST-CLI (NCT02060630) will re-address the question that was originally posed in the BASIL study, comparing the safety and efficacy of open versus endovascular revascularization in a randomized trial of patients eligible to undergo either. Unlike BASIL, however, BEST-CLI will include broader outcomes, such as need for re-intervention, quality of life measures, and cost effectiveness. The infusion of dexamethasone via the Bullfrog Micro-Infusion Device (Mercator MedSystems, Emeryville, CA, USA) is being studied as a means of reducing inflammation and restenosis in conjunction with other percutaneous interventions. The LIMBO trials are two active multi-center trials studying dexamethasone injection via the Bullfrog device after PTA or atherectomy, specifically in CLI patients. LIMBO-PTA (NCT02479555) is being carried out in Europe, and LIMBO-ATX (NCT02479620) in the US.

## 4. Conclusions

CLI is an important cause of morbidity and mortality among patients with PAD, for which percutaneous intervention is a reasonable first-line therapeutic approach. Numerous percutaneous approaches are available though no single device or combination of devices has demonstrated clear superiority. This lack of clarity is attributed to the limitations of the clinical evidence, including variation in outcomes that have been studied across clinical trials, the paucity of studies exclusively looking at patients with CLI, the inconsistent administration and the documentation of proper wound care following intervention, and a lack of direct comparisons of devices. Additionally, many clinical trials have been conducted at a single center or only a few centers by experienced operators, limiting their generalizability to the broader population. Ideally, ongoing multicenter randomized trials, including BEST-CLI, will significantly add to our knowledge base and help to guide future patient care.

Multidisciplinary care of CLI remains the cornerstone of therapy. In addition to physicians who are able to provide medical, percutaneous, and surgical therapies, this team also comprises wound care specialists, home health aides, and podiatrists, among others to ensure that an individual patient receives optimal care.
